# Pharmacokinetics of Edoxaban 15 mg in Very Elderly Patients with Nonvalvular Atrial Fibrillation: A Subanalysis of the ELDERCARE-AF Study

**DOI:** 10.1055/s-0044-1785511

**Published:** 2024-04-19

**Authors:** Takeshi Yamashita, Yoshiyuki Igawa, Masayuki Fukuzawa, Takuya Hayashi, Stefanie Hennig, Ken Okumura

**Affiliations:** 1 Department of Cardiovascular Medicine, The Cardiovascular Institute, Tokyo, Japan; 2 Quantitative Clinical Pharmacology Department, Daiichi Sankyo Co., Ltd., Tokyo, Japan; 3 Japan Business Unit, Primary Medical Science Department, Cardiovascular Group, Daiichi Sankyo Co., Ltd., Tokyo, Japan; 4 Data Analysis Group, Data Intelligence Department, Global DX, Daiichi Sankyo Co., Ltd., Tokyo, Japan; 5 Certara, Inc., Princeton, New Jersey, United States; 6 Division of Cardiology, Saiseikai Kumamoto Hospital, Kumamoto, Japan

**Keywords:** edoxaban, nonvalvular atrial fibrillation, elderly, pharmacokinetics

## Abstract

**Background** We evaluated the pharmacokinetics (PK) of low-dose (15 mg) edoxaban in very elderly patients (≥80 years) with nonvalvular atrial fibrillation (NVAF) and high bleeding risk.

**Methods** This subanalysis of the phase 3, randomized, double-blind, placebo-controlled, multicenter ELDERCARE-AF study evaluated edoxaban plasma concentrations and compared them with the Japanese population of the ENGAGE AF-TIMI 48 and Japanese severe renal impairment (SRI) studies.

**Results** The PK analysis population included 451 patients, 53.8% of whom concomitantly used antiplatelet drugs, 41.0% had SRI, and 38.0% had low body weight. Edoxaban plasma concentrations at trough and 1 to 3 hours post-dose in ELDERCARE-AF were 17.3 ± 13.9 ( *n* = 427) and 93.3 ± 57.8 ng/mL ( *n* = 447), respectively. These values were slightly higher than the 15 mg group in ENGAGE AF-TIMI 48 ( *n* = 79; 12.4 ± 12.1 and *n* = 115; 78.7 ± 45.0 ng/mL, respectively), lower than the ENGAGE AF-TIMI 48 high-dose reduced to 30 mg group ( *n* = 83; 25.1 ± 36.6 and *n* = 111; 150 ± 91.6 ng/mL, respectively), but similar to the Japanese SRI study ( *n* = 39; 18.4 ± 11.2 and *n* = 40; 96.8 ± 48.3 ng/mL, respectively). ELDERCARE-AF patients with SRI and low body weight (≤45 kg) had higher concentrations than those without, and those taking antiplatelet drugs had lower concentrations than those who were not.

**Conclusion** PK data support edoxaban 15 mg once daily for very elderly NVAF patients with high bleeding risk, with caution for patients with SRI and/or low body weight.

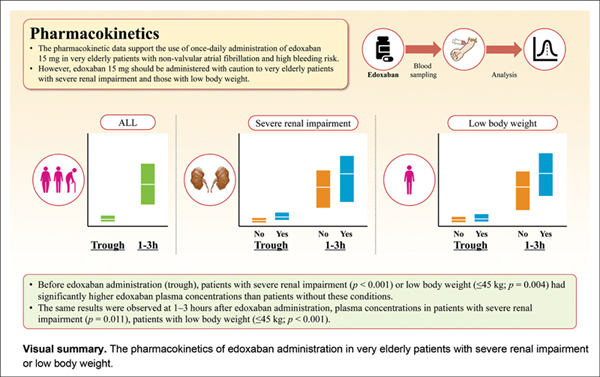

## Introduction

Atrial fibrillation (AF) is the most common sustained cardiac arrhythmia, and it increases in frequency with age. 1 AF has become an important health care problem globally, including in Japan, 2 specifically because of the increased stroke risk. 3 4

Edoxaban is usually taken at a dose of 60 mg once daily. The dosage is reduced to 30 mg if a patient has a low body weight (≤60 kg), has low creatinine clearance (CrCl ≤ 50 mL/min), or takes a concomitant P-glycoprotein (P-gp) inhibitor. 5 The Edoxaban Low-Dose for EldeR CARE AF patients (ELDERCARE-AF) study 6 was a randomized placebo-controlled study in which the efficacy and safety of edoxaban 15 mg once daily versus placebo were evaluated in Japanese patients with nonvalvular AF (NVAF) aged ≥80 years who were ineligible for standard oral anticoagulant therapy. In the ELDERCARE-AF population, we reported that the annual stroke or systemic embolism rates were 2.3% with edoxaban and 6.7% with placebo, major bleeding rates were 3.3 and 1.8%, and intracranial hemorrhage rates were 0.3 and 0.6%, respectively. 7 Based on the available evidence, edoxaban 15 mg has been approved in some Asian countries, including Japan, for stroke prevention in very elderly patients (≥80 years) with NVAF and bleeding risk. 7

A previous Japanese severe renal impairment (SRI) study evaluated the plasma concentration of edoxaban 15 mg in patients with NVAF with SRI (CrCl ≥15 to <30 mL/min) and that of edoxaban 30 to 60 mg in patients with normal renal function or mild renal impairment (CrCl ≥ 50 mL/min) during 12 weeks of treatment. 8 The Effective Anticoagulation with Factor Xa Next Generation in Atrial Fibrillation-Thrombolysis in Myocardial Infarction 48 (ENGAGE AF-TIMI 48) 9 trial compared two dose regimens of edoxaban (high dose: 60 mg reduced to 30 mg; low dose: 30 mg reduced to 15 mg) with warfarin in patients with AF aged ≥21 years. However, the pharmacokinetics (PK) of 15 mg edoxaban, which is administered to very elderly NVAF patients with high bleeding risk such as those included in the ELDERCARE-AF study, 6 remains unclear.

This prespecified subanalysis of the ELDERCARE-AF study aimed to evaluate the PK of edoxaban 15 mg once daily in NVAF patients aged ≥80 years. The PK results were also compared with those of the previous Japanese SRI study 8 and the Japanese population of ENGAGE AF-TIMI 48. 9

## Methods

### Study Design, Ethics, Randomization, and Intervention

The study design and detailed methodology of the ELDERCARE-AF study have been previously published. 6 Briefly, ELDERCARE-AF was a phase 3, randomized, double-blind, placebo-controlled, parallel-group, multicenter, event-driven, superiority trial conducted in Japan. The institutional review board at each study site approved the study protocol. The study conduct complied with the standards specified in the Pharmaceutical and Medical Devices Act, the Ministerial Ordinance on Good Clinical Practice for Drugs, and the Declaration of Helsinki. In addition, banking of clinical specimens for genomic and genetic analysis and research using these specimens was performed following the Ethical Guidelines for Human Genome/Gene Analysis Research and Ethical Guidelines for Clinical Research (only at study sites where genomic and genetic analysis or banking was approved).

Eligible patients were randomly assigned in a 1:1 ratio to receive 15 mg edoxaban or placebo once daily. The randomization scheme was permuted in blocks of four, and patients were stratified according to their CHADS _2_ scores (congestive heart failure, hypertension, age, diabetes mellitus, and previous stroke/transient ischemic attack; 2 points or ≥3 points). The patients, investigators, and sponsor were unaware of the trial group assignments to ensure blinding. Study drug administration was continued until the end-of-study drug administration test, which was performed within 60 days after the end of the study.

### Patients

The eligibility criteria have been published previously. 7 Briefly, eligible patients were aged ≥80 years, had a history of NVAF documented by electrocardiogram or monitor recording within 1 year of consent, had a CHADS _2_ score of ≥2, were not eligible to receive standard oral anticoagulant doses (i.e., warfarin, dabigatran, rivaroxaban, apixaban, or edoxaban), and presented with one or more of the five bleeding risks: SRI (CrCl ≥15 to <30 mL/min), a history of bleeding in a critical organ, low body weight (≤45 kg), chronic use of acidic nonsteroidal anti-inflammatory drugs (NSAIDs), and use of antiplatelet drugs. More information on the eligibility criteria can be found in the main ELDERCARE-AF paper. 7

A detailed list of exclusion criteria has been published. 7 Patients with transient AF due to reversible disorders; treated with other oral anticoagulants within 8 weeks of study initiation; who had severe bleeding or ischemic events within 30 days of randomization; with severe infections, uncontrolled hypertension, malignancies, severe heart disease, severe hepatic or kidney disorder; for whom edoxaban was contraindicated, including patients with CrCl <15 mL/min and with a high risk of bleeding, such as active bleeding, unresolved peptic ulcer, hemoglobin <9 g/dL or platelet count <10 × 10 ^4^ /µL, and hemorrhagic diseases; and who were considered ineligible by the investigator were excluded.

The details of the Japanese SRI 8 and ENGAGE AF-TIMI 48 studies have been published. 9 10

### PK Endpoints

As reported by Matsushima et al, edoxaban plasma concentrations were analyzed using a validated liquid chromatography-tandem mass spectrometry method. 11 Internal standards were mixed with plasma samples and extracted using solid-phase extraction. Gradient chromatography was performed with ammonium acetate and methanol as mobile phases. The high-performance liquid chromatography column used was Agilent Zorbax Eclipse XDB Phenyl. The assay had a lower limit of quantitation of 0.764 ng/mL and validated calibration curves ranging from 0.792 to 382 ng/mL. Edoxaban intra- and interassay precisions were ≤11.0% and ≤8.8%, respectively. 11 The PK endpoint was edoxaban plasma concentration at Week 8 during the study treatment period (Visit 3). Further details of the PK endpoint sample collection are available in the “Data/Sample Collection” section.

### Data/Sample Collection

The timing of the blood sampling at Week 8 of the study (Visit 3) was the trough time before the study drug administration (pre-dose). Blood samples were also collected at 1 to 3 hours after drug administration and (for patients who were willing to cooperate) at 4 to 8 hours after drug administration. Trough samples were drawn on the same day as the peak sample. Data on dosing times 1 and 2 days prior to sampling were collected using case report forms (CRFs). CRFs prepared for each patient were entered electronically using a validated electronic data collection system for preparing CRFs. Samples were processed, and measurements were obtained immediately upon sample receipt at central laboratories. Plasma concentration (PK endpoint) was measured by liquid chromatography-tandem mass spectrometry at Q 2 Solutions, Durham, North Carolina, United States.

### Sample Size Rationale and Calculations

Detailed information regarding the target sample size calculations is provided in the ELDERCARE-AF rationale and design article. 6 ELDERCARE-AF was an event-driven study, and it was considered that changes in the total number of patients could occur as necessary depending on the number of target events collected. The plan was to collect data for 65 primary endpoint events in the intention-to-treat population between randomization and the final follow-up examination. Under the conditions that the annual incidence in the placebo group was 5% per year, the hazard ratio of edoxaban relative to the placebo group was 0.5, the two-tailed significance level was 5%, and the power was 80%. The estimated number of patients to be enrolled in each group was approximately 400.

### Statistical Analysis

The PK analysis set consisted of all patients randomly assigned to receive edoxaban without major protocol deviations affecting PK and with at least one available edoxaban plasma concentration measurement.

For PK analysis, edoxaban concentrations in each study are reported using summary statistics. Likewise, the observed edoxaban plasma concentrations are presented using summary statistics by bleeding risk subgroups (i.e., SRI, a history of bleeding in a critical organ, low body weight [≤45 kg], chronic use of acidic NSAIDs, use of antiplatelet drugs). The plasma concentrations were compared using the Student's *t* -test across subgroups categorized by dose-adjustment factors. Additionally, external validation of a previously developed population PK (PopPK) model was performed, and the details are provided in the Supplementary Materials (available in the online version). Model-predicted exposure metrics were then compared by patient status.

For any given time point, unavailable data were considered missing for that time point. Missing data were not replaced by estimates or calculations for missing data. Individual CrCl values were calculated using the Cockcroft–Gault formula, and baseline CrCl was used for the analysis. Observed edoxaban plasma concentrations were summarized and presented across the overall population and stratified by variables of interest.

## Results

### Patient Disposition

Between August 5, 2016 and November 5, 2019, 1,086 patients from 164 institutions were enrolled, and 102 were excluded; 984 were randomly assigned (edoxaban 15 mg, *n* = 492; placebo, *n* = 492). The PK analysis population consisted of 451 patients in the edoxaban 15 mg group. The date of the last patient follow-up was December 27, 2019. The median (interquartile range) trial duration was 466.0 (293.5–708.0) days.

### Patient Characteristics

Patient baseline characteristics have been previously reported 7 and were considered well-balanced between groups. The baseline characteristics of the patients are shown in Table 1 . In the total population and the PK analysis set of ELDERCARE-AF, 7 the mean age was 86.6 and 86.7 years, 57.4 and 56.3% of patients were women, the mean weight was 50.6 and 50.8 kg, and the mean CrCl was 36.3 and 36.7 mL/min, respectively. Overall, 47.1 and 49.4% of patients had paroxysmal AF, 52.9 and 50.6% had nonparoxysmal AF, 43.0 and 40.8% had a history of oral anticoagulant treatment, and 7.6 and 6.0% concomitantly received at least one P-gp inhibitor, respectively. The mean CHADS _2_ score was 3.1 and 3.0, and the mean HAS-BLED score was 2.3 and 2.3, respectively. Overall, the most common risk factors for bleeding were concomitant use of one antiplatelet drug (53.8%), followed by SRI (41.0%), low body weight (38.0%), continuous use of acidic NSAIDs (32.2%), and a history of bleeding in a critical area or organ (22.6%). According to the frailty assessment, 40.9 and 37.3% of patients were frail, and 55.1 and 61.0% were robust or pre-frail, respectively.

**Table 1:** Demographic and clinical characteristics of patients

	Edoxaban, 15 mg ( *N* = 451)	Placebo ( *N* = 492)
Age, y, mean ± SD	86.7 ± 4.3	86.4 ± 4.3
Distribution, *n* (%)		
≤85 y	200 (44.3)	229 (46.5)
> 85 y	251 (55.7)	263 (53.5)
Male sex, *n* (%)	197 (43.7)	207 (42.1)
Type of atrial fibrillation		
Nonparoxysmal	228 (50.6)	266 (54.1)
Paroxysmal	223 (49.4)	226 (45.9)
Weight, kg	50.8 ± 10.6	50.6 ± 11.1
Body mass index a	22.1 ± 3.4	22.2 ± 3.8
Creatinine clearance, mean ± SD	36.7 ± 14.3	36.2 ± 14.5
Distribution, *n* (%)		
≤50 mL/min	376 (83.4)	408 (82.9)
> 50 mL/min	75 (16.6)	84 (17.1)
CHADS _2_ score b		
Mean score	3.0 ± 1.1	3.1 ± 1.1
Distribution, *n* (%)		
2	172 (38.1)	182 (37.0)
≥3	279 (61.9)	310 (63.0)
Components, *n* (%)		
Age ≥75 y	451 (100.0)	492 (100.0)
Previous stroke or transient ischemic attack	95 (21.1)	126 (25.6)
Congestive heart failure	223 (51.7)	274 (55.7)
Diabetes mellitus	106 (23.5)	110 (22.4)
Hypertension	378 (83.8)	398 (80.9)
CHA _2_ DS _2_ -VASc score c	4.8 ± 1.2	5.0 ± 1.3
HAS-BLED score d	2.3 ± 0.9	2.4 ± 0.9
Coronary artery disease	118 (26.2)	127 (25.8)
Dementia	63 (14.0)	90 (18.3)
History of falling within past year	139 (30.8)	186 (37.8)
Frailty category e		
Robust or pre-frail	275 (61.0)	253 (51.4)
Frail	168 (37.3)	217 (44.1)
Could not be evaluated	5 (1.11)	10 (2.0)
Missing data	3 (0.7)	12 (2.4)
History of oral anticoagulant therapy		
Yes	184 (40.8)	216 (43.9)
Warfarin	101 (22.4)	128 (26.0)
Direct oral anticoagulants f	109 (24.2)	127 (25.8)
Unknown	0 (0.0)	2 (0.4)
No	267 (59.2)	276 (56.1)
Concomitant Use of P-gp inhibitors	27 (6.0)	46 (9.3)
Number of concomitant medications		
Minimum	0	1
Median	9	9
Maximum	24	25

Abbreviations: P-gp, P-glycoprotein; PK, pharmacokinetics; SD, standard deviation.

Note: Plus–minus values are mean ± SD. Percentages may not total 100 because of rounding.

a The body mass index is the weight in kilograms divided by the square of the height in meters. Data were missing for 1 patient in the edoxaban group and 2 patients in the placebo group.

b CHADS _2_ scores range from 0 to 6, with higher scores indicating a greater risk of stroke. Previous stroke or transient ischemic attack is assigned 2 points, and congestive heart failure, hypertension, diabetes mellitus, and an age of ≥75 years are each assigned 1 point toward the total score.

c CHA _2_ DS _2_ -VASc scores range from 0 to 9, with higher scores indicating a greater risk of stroke. Previous stroke or transient ischemic attack and an age of ≥75 years are each assigned 2 points, and congestive heart failure, hypertension, diabetes mellitus, an age of 65–74 years, female sex, and history of vascular disease are each assigned 1 point toward the total score.

d HAS-BLED scores range from 0 to 9, with higher scores indicating a greater risk of bleeding. Abnormal renal or liver function are assigned 1 point each; the use of antiplatelet or nonsteroidal anti-inflammatory drugs or alcohol concomitantly is assigned 1 point each (for a total of 1 or 2 points), and hypertension, stroke, history of bleeding or a predisposition to bleeding, labile international normalized ratio, and elderly age (>65 years) are each assigned 1 point toward the total score.

e Frailty was assessed with the use of five measures of physical condition; a score of 0 indicated robust, a score of 1 or 2 indicated pre-frail, and a score of ≥3 indicated frail. Details of the assessment of frailty are provided in the Supplementary Materials (available in the online version).

f Direct oral anticoagulants included dabigatran, rivaroxaban, apixaban, and edoxaban.

### PK

**Fig. 1:**
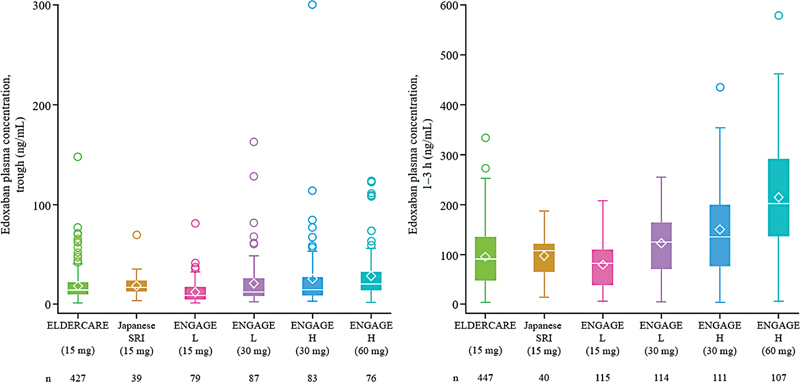
Comparison of edoxaban plasma concentration at trough and at 1 to 3 hours in the ELDERCARE-AF study, the Japanese SRI study, and the low- and high-dose groups of the ENGAGE AF-TIMI 48 study. Data from the ELDERCARE-AF and the Japanese SRI studies correspond to data from Week 8, and from the ENGAGE AF-TIMI 48 study correspond to data from Day 29. In the box and whisker plot, the box represents the interquartile range, and the line intersecting the box represents the median. The upper side of the box represents the 75th percentile + 1.5 × interquartile range. The lower side of the box represents the 25th percentile − 1.5 × interquartile range. The upper whisker point is the maximum observation below the upper side (75th percentile). The lower whisker point is the minimum observation above the lower side of the box (25th percentile). The lozenge represents the mean. The data points above the upper whisker indicate the outliers. h, hour; H, high-dose; L, low-dose; SRI, severe renal impairment.

The mean (±standard deviation) edoxaban plasma concentrations at trough and at 1 to 3 hours after administration in ELDERCARE-AF were 17.3 ± 13.9 ( *n* = 427) and 93.3 ± 57.8 ng/mL ( *n* = 447), respectively ( Fig. 1 , Supplementary Table S1 , available in the online version). When compared with the concentrations in the Japanese population of ENGAGE AF-TIMI 48, they were both slightly higher than those in the 15 mg group ( *n* = 79; 12.4 ± 12.1 and *n* = 115; 78.7 ± 45.0 ng/mL, respectively) and lower than those in the high-dose 60 mg group ( *n* = 76; 28.7 ± 27.2 and *n* = 107; 215 ± 119 ng/mL, respectively), high-dose 30 mg group ( *n* = 83; 25.1 ± 36.6 and *n* = 111; 150 ± 91.6 ng/mL, respectively), and low-dose 30 mg group ( *n* = 87; 20.8 ± 24.9 and *n* = 114; 122 ± 63.1 ng/mL). The concentrations were similar to those in the Japanese SRI study ( *n* = 39; 18.4 ± 11.2 and *n* = 40; 96.8 ± 48.3 ng/mL, respectively).

**Fig. 2:**
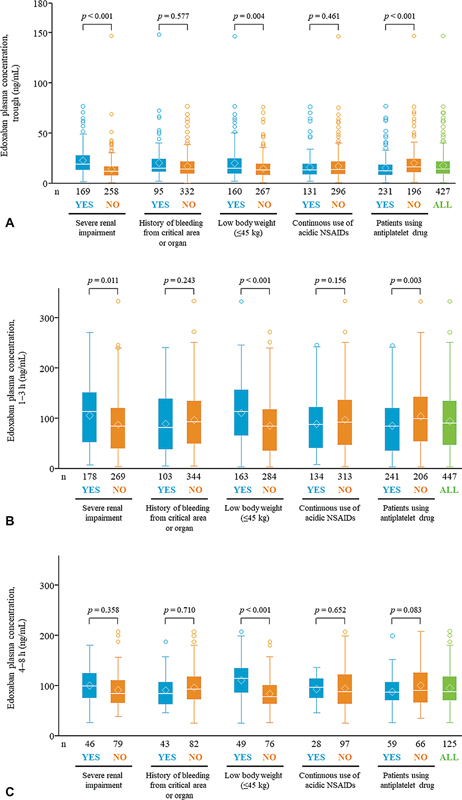
Edoxaban plasma concentration at ( **A** ) trough, ( **B** ) 1 to 3 hours, and ( **C** ) 4 to 8 hours post-dose in patients with or without bleeding risk factors. In the box and whisker plot, the box represents the interquartile range, and the line intersecting the box represents the median. The upper side of the box represents the 75th percentile + 1.5 × interquartile range. The lower side of the box represents the 25th percentile − 1.5 × interquartile range. The upper whisker point is the maximum observation below the upper side (75th percentile). The lower whisker point is the minimum observation above the lower side of the box (25th percentile). The lozenge represents the median. The data points above the upper whisker are outliers. h, hour; NSAIDs, nonsteroidal anti-inflammatory drugs.

The edoxaban plasma concentrations in ELDERCARE-AF patients with and without each risk factor were compared by bleeding risk subgroups ( Fig. 2 , Supplementary Table S2 , available in the online version). Before edoxaban administration (trough), patients with SRI ( *p* < 0.001) or low body weight (≤45 kg; *p* = 0.004) had higher edoxaban plasma concentrations than patients without these conditions. Plasma concentrations were lower in patients using antiplatelet drugs ( *p* < 0.001) than in patients not using antiplatelet drugs ( Fig. 2A ). At 1 to 3 hours after edoxaban administration, plasma concentrations in patients with SRI ( *p* = 0.011), patients with low body weight (≤45 kg; *p* < 0.001), and patients using antiplatelet drugs ( *p* = 0.003) showed similar patterns to those seen at trough ( Fig. 2B ). At 4 to 8 hours after edoxaban administration, the edoxaban plasma concentration was increased in patients with low body weight (≤45 kg; *p* < 0.001) compared with those with body weight >45 kg. Among patients treated and not treated with antiplatelet drugs, the mean weight was 54.12 and 46.99 kg, and the mean CrCl was 40.59 and 32.15 mL/min, respectively. No differences were observed in the other subgroups ( Fig. 2C ). The time variation in the edoxaban plasma concentration by each risk factor is shown in Supplementary Fig. S1 (available in the online version).

Currently, in clinical settings, edoxaban administration is subject to dose reduction by dose-adjustment factors of body weight ≤60 kg, CrCl ≤50 mL/min, or concomitant P-gp inhibitor. Although no dose reduction criteria were applied in this ELDERCARE-AF study, the plasma concentration of edoxaban with and without these dose-adjustment factors was evaluated to examine the impact of the dose reduction criteria on this study population. Plasma concentrations of edoxaban were higher in patients weighing ≤60 kg ( *p* < 0.001 at trough; *p* < 0.01 at 1–3 hours) and patients with CrCl ≤50 mL/min ( *p* < 0.001 at trough; *p* = 0.385 at 1–3 hours) than in patients who did not meet the respective criteria ( Supplementary Fig. S2 , Supplementary Table S3 [available in the online version]). Similar results were obtained for the exposure metrics when using the previously developed PopPK model, indicating that the existing model accurately describes the data of the ELDERCARE-AF study ( Supplementary Fig. S3 , available in the online version). A comparison of steady-state exposure (steady-state trough concentration [ *C*_min,ss_ ], steady-state peak concentration [ *C*_max,ss_ ], and steady-state area under the curve [AUC _ss_ ]) to edoxaban in the ELDERCARE-AF study by the dose-adjustment factors and the high-dose groups of ENGAGE AF-TIMI 48 is provided in Supplementary Fig. S4 (available in the online version). The exposures of all subpopulations in the ELDERCARE-AF study were lower than those of the high-dose group in ENGAGE AF-TIMI 48.

### Pharmacodynamics

The pharmacodynamics results are summarized in the Supplementary Materials (available in the online version).

## Discussion

In the present study, we evaluated the PK of edoxaban 15 mg in very elderly patients from the ELDERCARE-AF study 7 compared with the Japanese population of the ENGAGE AF-TIMI 48 9 and Japanese SRI 8 studies. Moreover, we determined the effects of bleeding risk factors on PK in this patient population.

The edoxaban plasma concentrations at trough and at 1 to 3 hours after administration in the ELDERCARE-AF study were slightly higher than in the Japanese 15 mg group in ENGAGE AF-TIMI 48 and lower than in the ENGAGE AF-TIMI 48 high-dose groups, but similar to those in the Japanese SRI study ( *n* = 40). Furthermore, the concentrations were higher in patients with SRI and low body weight in ELDERCARE-AF than in those without. In patients using antiplatelet drugs, the concentrations were lower than in those who were not.

In the Japanese population of ENGAGE AF-TIMI 48, the edoxaban concentration increased in association with lower body weight and impaired renal function. When comparing the PK values of edoxaban in very elderly ELDERCARE-AF patients with those in ENGAGE AF-TIMI 48, edoxaban 15 mg appears to be therapeutically effective for very elderly patients. This is based on the observation that the PK values were slightly higher than those in the Japanese 15 mg group of ENGAGE AF-TIMI 48, in which the low-dose group (30 or 15 mg) was noninferior to warfarin. 9 Although the edoxaban 15 mg dosage was not approved in Japan based on the ENGAGE AF-TIMI 48 study, it was subsequently approved based on the findings of the ELDERCARE-AF study in NVAF patients with older age and high bleeding risk. However, the patient background characteristics differed between the studies (mainly in bleeding risk), which may affect the risk–benefit balance of this edoxaban dose. Thus, caution is still required when administering edoxaban 15 mg to very elderly patients with SRI and/or low body weight because of increased exposure to edoxaban. However, in patients with very high bleeding risk, exposure to edoxaban 15 mg did not exceed that of the high-dose group (60 or 30 mg) from the Japanese population of ENGAGE AF-TIMI 48. 12 In the ELDERCARE-AF study, 7 edoxaban 15 mg numerically increased major bleeding events compared with placebo, but it did not increase intracranial hemorrhage or fatal bleeding. These findings support the feasibility of using this edoxaban dose for elderly patients with NVAF who have a high bleeding risk, as the increase in bleeding events was minimal.

The incidences of stroke/systemic embolic events and major hemorrhage in the ELDERCARE-AF study 7 were comparable with those in the subgroup analyses of other direct oral anticoagulants in elderly patients. 13 14 15 Although the present PK data show a slightly lower plasma concentration with edoxaban 15 mg than the plasma concentration observed with the usual edoxaban dose in the elderly population in the ENGAGE AF-TIMI 48 study, 16 the incidences of stroke/systemic embolic events and major bleeding were similar between the studies. These results support that edoxaban 15 mg once daily may be an appropriate regimen in very elderly AF patients with high bleeding risk.

This study was designed to include elderly patients for whom regular anticoagulant doses were deemed inappropriate. It was also designed not to exclude the relatively robust patients who did not meet the criteria for dose reduction. Therefore, the study population included patients with relatively high CrCl and body weight values, many of whom fulfilled the inclusion criteria of receiving treatment with long-term NSAIDs or antiplatelet agents. Fig. 2 indicates that edoxaban plasma concentrations at trough and at 1 to 3 hours post-dose were lower in patients with concomitant antiplatelet agents as a bleeding risk factor. These results could be explained by patient characteristics, such as body weight >60 kg and/or CrCl >50 mL/min ( Supplementary Fig. S4 , available in the online version). Given these considerations, administration of edoxaban 15 mg to patients with high CrCl and high body weight solely because of long-term antiplatelet use may raise concerns about inadequate efficacy in terms of lowering the edoxaban plasma concentration. Therefore, it may be necessary to discontinue antiplatelet agents before prescribing edoxaban 15 mg to patients who do not meet the dose reduction criteria and to consider administration of the usual dose by eliminating potential bleeding risk factors.

There are limitations associated with this subanalysis of the ELDERCARE-AF study. Only data from Japanese patients were included and analyzed. The sample size for this analysis was based on the main ELDERCARE-AF study, 7 and the sample size was not set to calculate PK data. Furthermore, the PK blood sampling was infrequent and was only performed a maximum of three times at 8 weeks post-dose (Visit 3). The small number of events limited the confirmation of the association between events and the plasma concentration of edoxaban. Finally, as the 15 mg edoxaban and low-dose 30 mg edoxaban regimens used in the ENGAGE AF-TIMI 48 study are not disclosed in public documents, some comparisons could not be made between these groups and the patients in the ELDERCARE-AF study.

In conclusion, based on the present PK analysis, edoxaban 15 mg once daily may offer an alternative risk–benefit balance in very elderly AF patients at high bleeding risk.

## Notes

**What is known about this topic?**

- Atrial fibrillation is a major global health care problem because of its association with an increased risk of stroke.
- Previous studies indicate the effectiveness of low doses of the anticoagulant medication edoxaban for nonvalvular atrial fibrillation in very elderly patients ineligible for standard oral anticoagulant therapy.
- However, the pharmacokinetics of low doses of edoxaban in very elderly patients with nonvalvular atrial fibrillation and high bleeding risk remains to be elucidated.

**What does this paper add?**

- At a dose of 15 mg, edoxaban plasma concentrations at trough and 1 to 3 hours post-dose were slightly higher than those of patients who received edoxaban 15 mg and lower than those in patients who received edoxaban 30 mg in the Japanese population of ENGAGE AF-TIMI 48 study. However, concentrations were similar to those of patients with severe renal impairment in another Japanese study.
- The pharmacokinetic data support the use of once-daily administration of edoxaban 15 mg in very elderly patients with nonvalvular atrial fibrillation and high bleeding risk.
- However, in patients who received edoxaban 15 mg with severe renal impairment and low body weight (≤45 kg), edoxaban plasma concentrations were higher than in those without these conditions. Therefore, edoxaban 15 mg should be administered with caution to very elderly patients with severe renal impairment and those with low body weight.
