# Supplementary material for: Pharmacokinetics of Edoxaban 15 mg in Very Elderly Patients with Nonvalvular Atrial Fibrillation: A Subanalysis of the ELDERCARE-AF Study
Source: Thromb Haemost. 2024 Apr 19;124(9):874–82. doi: 10.1055/s-0044-1785511 (PMC11349424; doi:10.1055/s-0044-1785511)
Supplement: Supplementary file 1 — Supplementary Material [file 10-1055-s-0044-1785511-s23040134.pdf]

Supplementary Materials

Supplementary Methods

The observed plasma concentrations at Week 8 during the study period (Visit 3) were used to evaluate the pharmacokinetic (PK) parameters in patients in the ELDERCARE-AF study using a previously established population PK (PopPK) model.[17] A numerical predictive check/visual predictive check approach was used, and standard goodness-of-fit plots were produced. Using the existing validated PopPK model for patients with nonvalvular atrial fibrillation, including Japanese patients with severe renal impairment,[17] individual empirical Bayes PK estimates for patients in ELDERCARE-AF were generated, as well as the individual edoxaban exposures at steady state (steady-state trough concentration [$C_{min,ss}$], steady-state peak concentration [$C_{max,ss}$], steady-state area under the curve [AUC_{ss}]).

Nonlinear mixed-effects modeling software (NONMEM) (version 7.4 or higher, ICON, Hanover, Maryland, United States) was used for the external validation analysis and empirical Bayesian estimation using first-order conditional estimation with interaction. Perl Speaks NONMEM (Department of Pharmacy, Uppsala University, Uppsala, Sweden) was used for model diagnostics, and R (version 4.0.0 or higher) was used for postprocessing of the results.

The pharmacodynamic endpoints were prothrombin time (PT), activated partial thromboplastin time (aPTT), D-dimer, and prothrombin fragments 1 + 2 (F1 + F2) at Week 8.

Supplementary Results

Changes in edoxaban plasma concentration by each risk factor for bleeding at trough, 1 to 3 hours, and 4 to 8 hours post-dose are shown in ►**Supplementary Fig. S1**.

►**Supplementary Fig. S2** shows the edoxaban plasma concentrations by dose-adjustment factors at trough and at 1 to 3 hours. The plasma concentrations of edoxaban were elevated in patients with body weight ≤60 kg and creatinine clearance (CrCl) ≤50 mL/min at trough and at 1 to 3 hours post-dose.

PopPK

Four patients had more than one missing dosing record and were excluded from the PopPK analysis, resulting in 1,012 evaluable PK samples from 447 patients in the ELDERCARE-AF study being included in the analysis.

An external predictive check confirmed that the existing PopPK model was applicable to the patient population in the ELDERCARE-AF study (►**Supplementary Fig. S3**). The figure indicates that the model predicts the central tendency of the edoxaban concentration–time data from the ELDERCARE-AF study.

►**Supplementary Fig. S4A** compares the $C_{min,ss}$ of edoxaban in patients from ELDERCARE-AF by dose-adjustment factors with that of edoxaban in the ENGAGE AF-TIMI 48. In the ELDERCARE-AF study, the median $C_{min,ss}$ was higher in the subgroup weighing ≤60 kg than in the subgroup weighing >60 kg. In addition, the median $C_{min,ss}$ was higher in the subgroup with CrCl ≤50 mL/min than in the subgroup with CrCl >50 mL/min. There was no difference in the $C_{min,ss}$ between the subgroup with and without P-glycoprotein inhibitors. The $C_{min,ss}$ for each subgroup of the ELDERCARE-AF study was lower than the exposure for the high-dose group in the ENGAGE AF-TIMI 48 study.

Similarly, in the ELDERCARE-AF study, the $C_{max,ss}$ was higher among patients weighing ≤60 kg versus >60 kg, and in those with CrCl ≤50 mL/min versus >50 mL/min. When compared with the high-dose groups of the ENGAGE AF-TIMI 48, the $C_{max,ss}$ was lower in every subgroup of the ELDERCARE-AF study (►**Supplementary Fig. S4B**). The findings for the AUC_{ss} were similar to those for the $C_{min,ss}$ and $C_{max,ss}$ (►**Supplementary Fig. S4C**). Higher AUC_{ss} values in the low body weight and low renal function groups of ELDERCARE-AF were seen, and the AUC_{ss} values in both dose groups of the ENGAGE AF-TIMI 48 were higher than all subgroups of the ELDERCARE-AF study.

Pharmacodynamics

►**Supplementary Table S4** summarizes the pharmacodynamic endpoints. The mean PTs at 1 to 3 and 4 to 8 hours post-dose increased compared with those at Visit 1 and trough in the edoxaban group. At the same time points, the mean ± standard deviation aPTTs remained similar in the placebo group (28.1 ± 3.5, 27.6 ± 2.5, and 27.6 ± 2.8 seconds but increased slightly at 4 to 8 hours post-dose in the edoxaban group (28.5 ± 3.0, 30.7 ± 3.8, and 31.1 ± 4.3 seconds, respectively.

 © 2024. The Author(s).

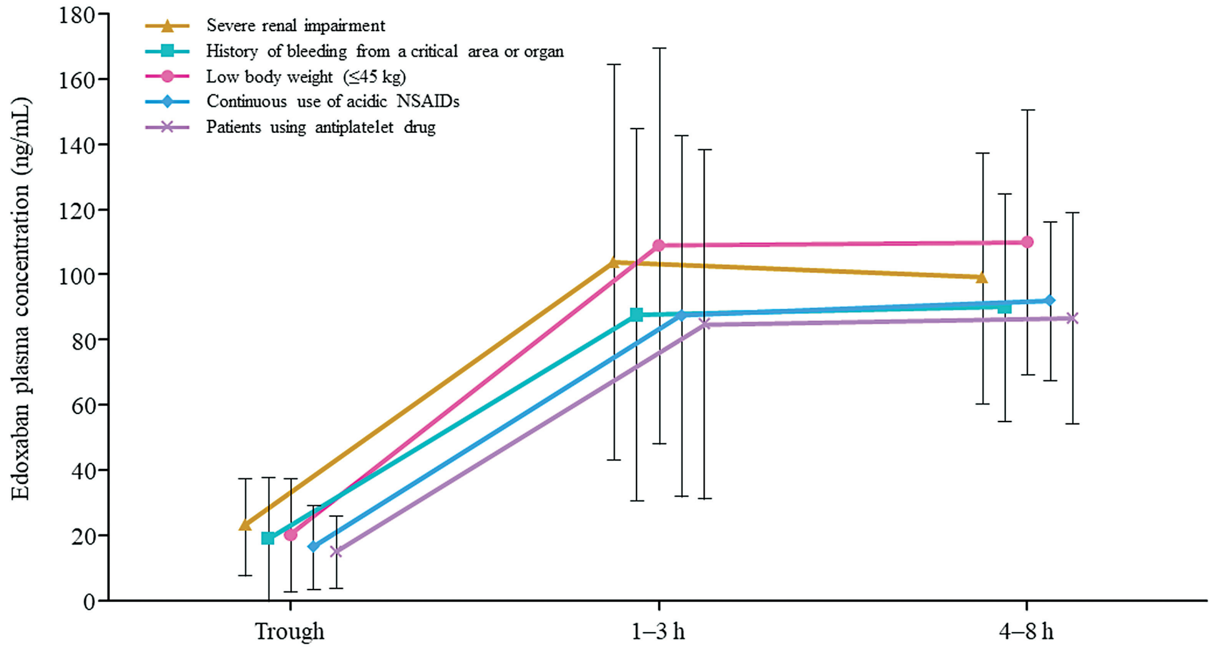

Supplementary Fig. S1 Change in edoxaban plasma concentration for each risk factor at trough, 1 to 3 hours, and 4 to 8 hours. Data are mean ± standard deviation. h, hour; NSAIDs, nonsteroidal anti-inflammatory drugs.

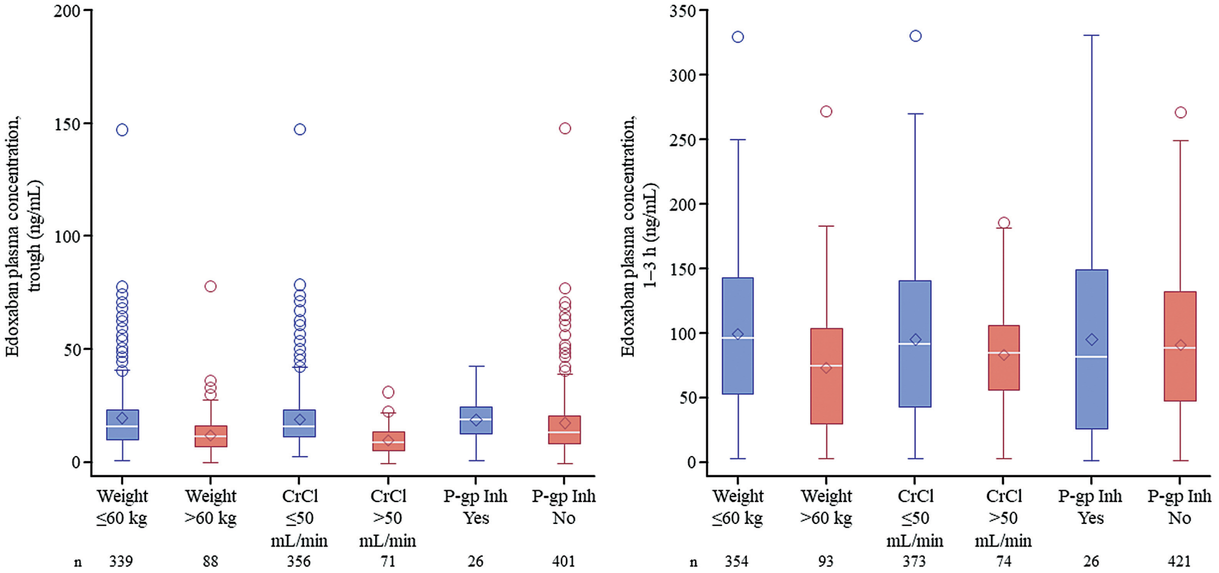

Supplementary Fig. S2 Edoxaban plasma concentration at trough and at 1 to 3 hours by dose-adjustment factors. In the box and whisker plot, the box represents the interquartile range, and the line intersecting the box represents the median. The upper side of the box represents the 75th percentile + 1.5 × interquartile range. The lower side of the box represents the 25th percentile − 1.5 × interquartile range. The upper whisker point is the maximum observation below the upper side (75th percentile). The lower whisker point is the minimum observation above the lower side of the box (25th percentile). The data points above the upper whisker are outliers. CrCl, creatinine clearance; P-gp Inh, P-glycoprotein inhibitor.

 © 2024. The Author(s).

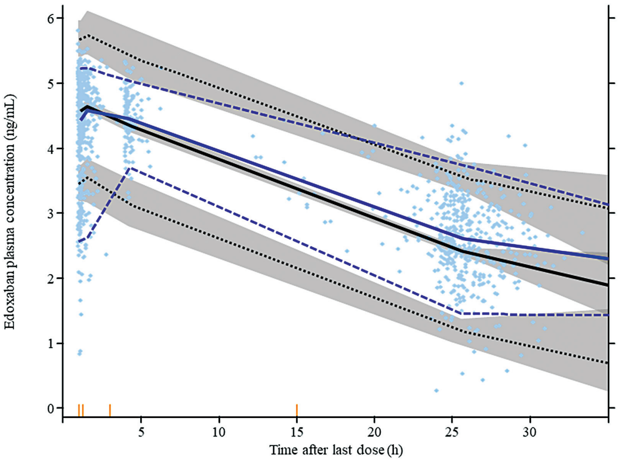

Supplementary Fig. S3 External predictive check of the population pharmacokinetics model to indicate the applicability of the existing population pharmacokinetics model to the patient population in ELDERCARE-AF. The blue dots are prediction-corrected observed concentrations after edoxaban administration in Study DU176b-C-J316 subjects; the blue lines are the 50th (solid), 5th (dashed), and 95th (dashed) percentiles of the observed concentrations; and the black lines are the 50th (solid), 5th (dashed), and 95th (dashed) percentiles of the simulations. The gray bands are the 95% prediction intervals for the corresponding black lines based on 1,000 simulations. h, hour.

 © 2024. The Author(s).

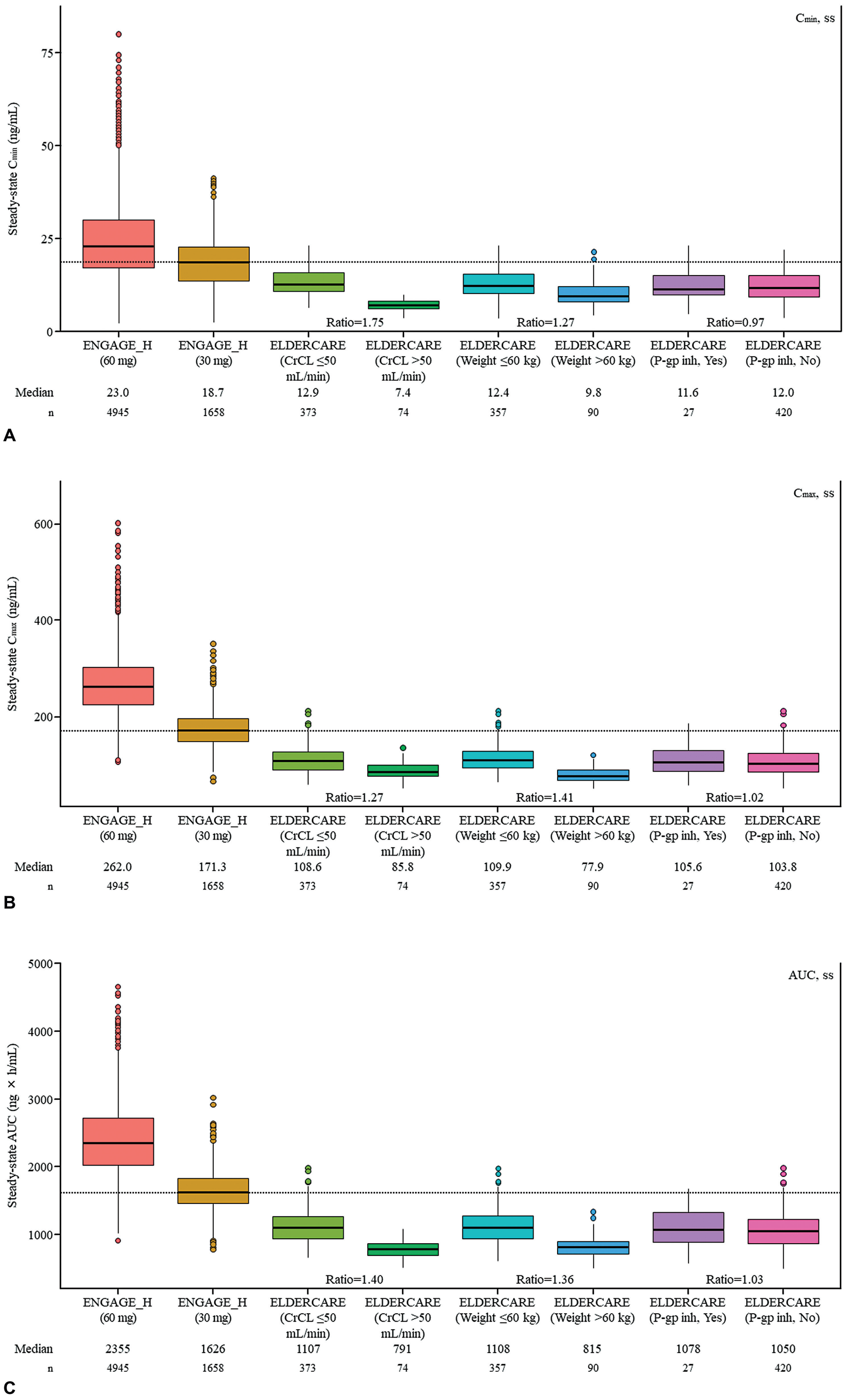

Supplementary Fig. S4 Comparison of steady-state exposure (**A**) $C_{min,ss}$, (**B**) $C_{max,ss}$, and (C) AUC_{ss} to edoxaban in the ELDERCARE-AF study by dose-adjustment factors and the high-dose groups of the ENGAGE AF-TIMI 48 study. In the box and whisker plot, the box represents the interquartile range, and the line intersecting the box represents the median. The upper side of the box represents the 75th percentile + 1.5 × interquartile range. The lower side of the box represents the 25th percentile − 1.5 × interquartile range. The upper whisker point is the maximum observation below the upper side (75th percentile). The lower whisker point is the minimum observation above the lower side of the box (25th percentile). The data points above the upper whisker are outliers. The dotted line indicates the median values for the ENGAGE AF-TIMI 48 high-dose group with dose-adjustment factors. AUC_{ss}, steady-state area under the curve; $C_{max,ss}$, steady-state peak concentration; $C_{min,ss}$, steady-state trough concentration; CrCl, creatinine clearance; P-gp Inh, P-glycoprotein inhibitor.

 © 2024. The Author(s).

Supplementary Table S1 Comparison of edoxaban plasma concentrations at trough and at 1 to 3 hours in the ELDERCARE-AF study, the Japanese SRI study, and the low- and high-dose groups of the ENGAGE AF-TIMI 48 study

		ELDERCARE	Japanese SRI	ENGAGE H (60 mg)	ENGAGE H (30 mg)	ENGAGE L (30 mg)	ENGAGE L (15 mg)
Trough	*n*	427	39	76	83	87	79
	Mean	17.3	18.4	28.7	25.1	20.8	12.4
	SD	13.9	11.2	27.2	36.6	24.9	12.1
	Median	13.6	16.7	20.1	14.7	11.7	8.7
1–3 h	*n*	447	40	107	111	114	115
	Mean	93.3	96.8	215	150	122	78.7
	SD	57.8	48.3	119	91.6	63.1	45.0
	Median	89.0	108	202	135	125	82.2

Abbreviations: H, high; L, low; SD, standard deviation; SRI, severe renal impairment.

Supplementary Table S2 Edoxaban plasma concentration at trough, at 1 to 3 hours, and 4 to 8 hours post-dose in patients with or without bleeding risk factors

		Severe renal impairment		History of bleeding from a critical area or organ		Low body weight ($\leq$45 kg)		Continuous use of acidic NSAIDs		Patients using antiplatelet drug		All
		Yes	No	Yes	No	Yes	No	Yes	No	Yes	No	
Trough	*n*	169	258	95	332	160	267	131	296	231	196	427
	Mean	22.7	13.8	19.0	16.8	20.2	15.6	16.5	17.7	14.9	20.2	17.3
	SD	14.9	12.0	19.0	12.0	17.4	11.0	12.9	14.3	10.9	16.3	13.9
	Median	18.8	11.4	13.5	13.6	15.0	13.2	13.4	13.7	12.0	16.0	13.6
	p-Value	$<$0.001		0.557		0.004		0.461		$<$0.001		
1–3 h	*n*	178	269	103	344	163	284	134	313	241	206	447
	Mean	104	86.1	87.8	94.9	109	84.1	87.5	95.8	85.0	103	93.3
	SD	60.7	54.7	57.0	58.0	60.8	53.9	55.4	58.7	53.7	60.9	57.8
	Median	112	83.2	79.7	91.1	112	83.1	85.7	90.3	83.2	98.0	89.0
	p-Value	0.011		0.243		$<$0.001		0.156		0.003		
4–8 h	*n*	46	79	43	82	49	76	28	97	59	66	125
	Mean	99.0	90.2	90.1	95.2	110	82.9	92.1	93.8	86.7	99.5	93.4
	SD	38.5	36.9	34.9	38.9	40.6	31.4	24.4	40.7	32.4	40.9	37.5
	Median	98.3	82.5	82.5	90.9	113	77.2	95.3	86.5	85.3	88.7	86.6
	p-Value	0.358		0.710		$<$0.001		0.652		0.083		

Abbreviations: NSAIDs, nonsteroidal anti-inflammatory drugs; SD, standard deviation.

 © 2024. The Author(s).

Supplementary Table S3 Edoxaban plasma concentrations at trough and at 1 to 3 hours by dose-adjustment factors

		Body weight		Creatinine clearance		P-gp inhibitor		All
		≤60 kg	>60 kg	≤50 mL/min	>50 mL/min	Yes	No	
Trough	*n*	339	88	356	71	26	401	427
	Mean	18.5	12.9	18.9	9.2	19.9	17.1	17.3
	SD	14.5	10.0	14.5	5.8	10.6	14.1	13.9
	Median	14.6	10.7	15.1	8.1	18.6	13.5	13.6
	p-Value	<0.001		<0.001		0.158		
1–3 hours	*n*	354	93	373	74	26	421	447
	Mean	98.9	71.8	95.4	82.4	94.7	93.2	93.3
	SD	59.0	47.6	60.1	43.2	82.4	56.1	57.8
	Median	94.8	74.1	90.8	83.6	81.8	89.1	89.0
	p-Value	<0.001		0.358		0.237		

Abbreviations: P-gp, P-glycoprotein; SD, standard deviation.

Supplementary Table S4 Pharmacodynamic endpoints

	Placebo				Edoxaban 15 mg			
	Visit 1 ($N=492$)	Visit 3 (Week 8)			Visit 1 ($N=490$)	Visit 3 (Week 8)		
		Trough ($n=448$)	1–3 hours ($n=449$)	4–8 hours ($n=127$)		Trough ($n=449$)	1–3 hours ($n=446$)	4–8 hours ($n=131$)
Prothrombin time (s)	12.8 ± 1.0	12.8 ± 1.2	13.0 ± 1.0	13.2 ± 1.2	12.8 ± 1.0	13.2 ± 1.2	15.4 ± 2.0	15.5 ± 1.8
aPTT (s)	27.6 ± 2.3	28.1 ± 3.5	27.6 ± 2.5	27.6 ± 2.8	27.5 ± 2.8	28.5 ± 3.0	30.7 ± 3.8	31.1 ± 4.3
D-dimer (µg/mL)	2.2 ± 2.8	–	2.1 ± 2.9	–	2.1 ± 2.9	–	1.0 ± 1.5	–
F1 + F2 (pmol/L)	454.7 ± 203.1	–	469.0 ± 204.9	–	463.9 ± 205.8	–	288.1 ± 134.0	–

Abbreviations: aPTT, activated partial thromboplastin time; F1 + F2, prothrombin fragments 1 + 2.
Note: Data are mean ± standard deviation.

 © 2024. The Author(s).
